# PANDORA-Seq Unveils the Hidden Small Non-Coding RNA Landscape in Hypopharyngeal Carcinoma

**DOI:** 10.3390/ijms26135972

**Published:** 2025-06-21

**Authors:** Miaoyan Pu, Luyu Shi, Haiyu Ma, Chuntao Tao, Ying Zhang, Youquan Bu, Junhong Ye

**Affiliations:** 1 Department of Biochemistry and Molecular Biology, College of Basic Medical Sciences, Chongqing Medical University, Chongqing 400016, China; 2023110030@stu.cqmu.edu.cn (M.P.); 2023110032@stu.cqmu.edu.cn (L.S.); 2022420030@stu.cqmu.edu.cn (H.M.); taochuntao1996@stu.cqmu.edu.cn (C.T.); zhangying@cqmu.edu.cn (Y.Z.); 2 Molecular Medicine and Cancer Research Center, Chongqing Medical University, Chongqing 400016, China

**Keywords:** hypopharyngeal carcinoma, small non-coding RNA, PANDORA-seq, tRNA-derived small RNA, rRNA-derived small RNA

## Abstract

Hypopharyngeal carcinoma is a highly aggressive malignancy in the head and neck region with poor prognosis due to challenges in early diagnosis, high invasiveness, recurrence rate, and metastatic potential. Small non-coding RNAs (sncRNAs) play crucial roles in tumorigenesis and progression and hold potential as clinical diagnostic biomarkers and therapeutic targets. However, the ability of traditional RNA-sequencing technologies to detect modified sncRNAs is limited, potentially leading to the failure to accurately identify some functionally relevant sncRNAs. In this study, we employed PANDORA-seq technology for the first time to systematically profile sncRNA expression in cancerous and adjacent normal tissues from five patients with hypopharyngeal carcinoma. Our results revealed dynamic changes in sncRNA expression in hypopharyngeal carcinoma tissues and found 4798 significantly differentially expressed sncRNAs. Among these, differentially expressed miRNAs and tsRNAs were primarily involved in key signaling pathways, including MAPK, FoxO, and TGF-β. Additionally, we validated the differential expression of eight sncRNAs in hypopharyngeal carcinoma tissues, which may represent potential diagnostic biomarkers and therapeutic targets. This study lays the foundation for the application of PANDORA-seq technology in human cancers and offers new directions for exploring the underlying molecular mechanisms of hypopharyngeal carcinoma and potential targets for its clinical diagnosis and treatment.

## 1. Introduction

Hypopharyngeal carcinoma is an aggressive head and neck squamous cell carcinoma that originates from the mucosal epithelium of the piriform sinus, postcricoid area, and posterior pharyngeal wall [1,2]. Owing to its concealed anatomical location [3], nonspecific early symptoms, and the lack of effective clinical diagnostic biomarkers, approximately 80% of patients are diagnosed at an advanced stage, with a high rate of lymph node metastasis (60–80%) [4,5,6]. Consequently, hypopharyngeal carcinoma is one of the most malignant head and neck tumors. The treatment strategies for hypopharyngeal carcinoma primarily depend on disease staging and patient health status, including surgery, radiotherapy, targeted therapy, and immunotherapy. The current focus of treatment strategies is on enhancing local and regional tumor control while minimizing functional impairment, thereby improving the quality of life for patients. For example, targeted therapies against PD-1 and PD-L1, such as pembrolizumab and nivolumab, have demonstrated promising efficacy in clinical trials for head and neck tumors and have been approved by the FDA for the treatment of recurrent or metastatic head and neck cancer [7]. However, due to its strong invasiveness, high recurrence rate, and propensity for distant metastasis, the 5-year survival rate remains low at 30–35% [4,6,8]. Therefore, in-depth investigations of the molecular mechanisms underlying hypopharyngeal carcinoma development, the identification of specific molecular markers, and the exploration of potential therapeutic targets are of great significance for improving early diagnosis rates, optimizing treatment strategies, and improving patient prognosis.

Small non-coding RNAs (sncRNAs) play critical roles in gene expression regulation and tumorigenesis. In addition to the well-known microRNAs (miRNAs), sncRNAs also include tRNA-derived small RNAs (tsRNAs), rRNA-derived small RNAs (rsRNAs), and YRNA-derived small RNAs (ysRNAs). These sncRNAs are not only involved in gene expression regulation [9,10,11] but also serve as potential diagnostic and prognostic tumor biomarkers [12,13,14] and hold promise as therapeutic targets for cancer treatment [15,16,17,18]. In recent years, traditional RNA sequencing (RNA-seq) techniques have been used to analyze the sncRNA expression profiles of various malignant tumors, including lung, liver, and gastric cancers [14,19,20,21]. Lu et al. [15] employed traditional RNA-seq to obtain the tsRNA expression profiles of tumor and adjacent normal tissues from 10 colorectal cancer patients. They further confirmed that tRF-3022b, tRF-3030b, and tRF-5008b were significantly upregulated in tumor tissues using qRT-PCR. The area under the curve (AUC) values of these three tsRNAs in distinguishing cancer patients from healthy individuals were 0.7684, 0.7485, and 0.6921 for tRF-3022b, tRF-3030b, and tRF-5008b, respectively. Knockdown of these tsRNAs was shown to arrest the cell cycle and induce apoptosis in colorectal cancer cells. These findings further confirm the significant regulatory roles of sncRNAs in tumorigenesis and their potential as diagnostic biomarkers and therapeutic targets. However, compared with other cancers, research on sncRNAs in hypopharyngeal carcinoma remains relatively limited, and systematic studies on the expression characteristics and functional mechanisms of sncRNAs in hypopharyngeal carcinoma are still in the early stages. Therefore, this study aimed to systematically characterize the sncRNA expression profiles in hypopharyngeal carcinoma, identify significantly differentially expressed sncRNAs, and explore their potential roles in the development of hypopharyngeal carcinoma, thereby providing new targets and a theoretical basis for improving the diagnosis and treatment of hypopharyngeal carcinoma.

Traditional RNA-seq has technical limitations in detecting non-canonical sncRNAs. First, many non-canonical sncRNAs possess unique terminal structures, such as 5′-OH, 3′-P, and 2′,3′-cP, which can hinder cDNA library construction. Second, chemical modifications on sncRNAs (such as m^1^A, m^3^C, m^1^G, and m^2^_2_G) may interfere with the reverse transcription process during library construction, resulting in inaccurate profiling of their true expression levels by traditional RNA-seq [22,23,24,25]. To overcome these challenges, we developed a novel sncRNA sequencing technology: panoramic RNA display by overcoming RNA modification aborted sequencing (PANDORA-seq). Unlike traditional RNA-seq, PANDORA-seq employs a combination of α-ketoglutarate-dependent hydroxylase (Alkb) and T4 polynucleotide kinase (T4PNK) enzymes to remove RNA modifications and unique terminal structures that impede cDNA library construction. Specifically, Alkb can remove methylated modifications, while T4PNK can convert 5′-OH to 5′-P and transform 3′-P and 2′,3′-cP to 3′-OH, thereby facilitating adapter ligation during library construction. Thus, PANDORA-seq provides a more comprehensive and accurate panoramic view of sncRNAs than traditional RNA-seq [23,26].

In this study, we used PANDORA-seq for the first time to systematically analyze the sncRNA expression profile of hypopharyngeal carcinoma and identified a series of differentially expressed sncRNAs. We further explored their potential roles in the development of hypopharyngeal carcinoma, thereby providing new directions for research into the molecular mechanisms and clinical applications of this disease.

## 2. Results

### 2.1. PANDORA-Seq Reveals a More Comprehensive Panoramic View of sncRNAs

**Figure 1:**
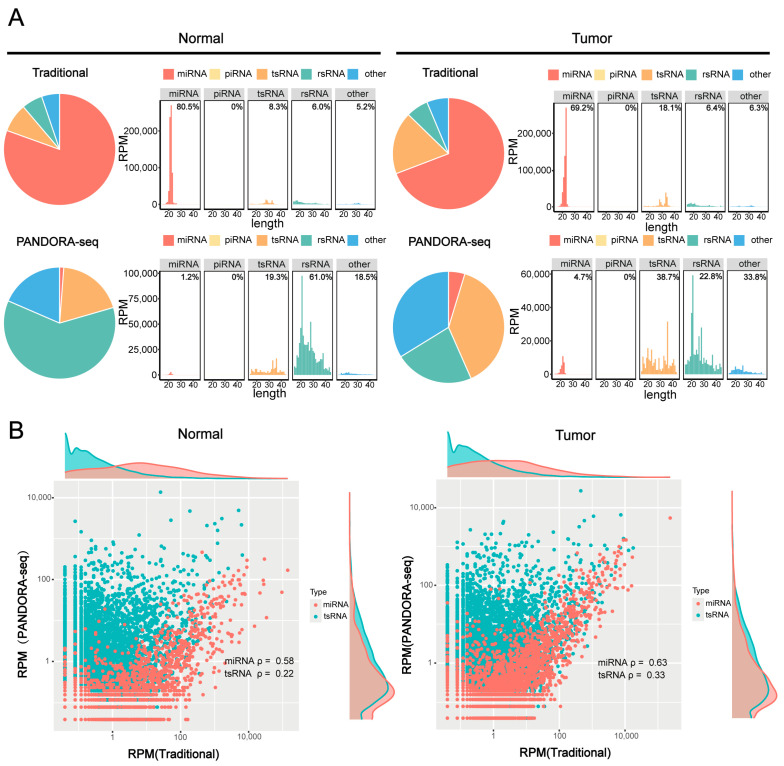
Comparison of sncRNA expression in hypopharyngeal carcinoma tissues using PANDORA-seq and traditional-seq. (**A**) Changes in the proportion of different sncRNA types in hypopharyngeal carcinoma tissues and adjacent normal tissues before and after treatment with α-ketoglutarate-dependent hydroxylase (Alkb) and T4 polynucleotide kinase (T4PNK) enzymes. (**B**) Correlation analysis of tsRNA and miRNA read proportions in hypopharyngeal carcinoma tissues and adjacent normal tissues before and after treatment with Alkb and T4PNK enzymes.

To validate the efficacy of PANDORA-seq technology in detecting sncRNAs in tumors, we compared sncRNA profiles in hypopharyngeal carcinoma tissues and adjacent normal tissues before and after enzymatic treatment to validate the efficacy of PANDORA-seq. Traditional RNA-seq mainly detects miRNAs, while Alkb and T4PNK treatment significantly increases the proportions of tsRNA and rsRNA detected. PANDORA-seq effectively identifies sncRNAs that are elusive to traditional sequencing methods (Figure 1A). Further analysis of the correlation between tsRNAs and miRNAs before and after enzymatic treatment (Figure 1B) revealed that the correlation of tsRNAs was significantly weaker than that of miRNAs. Specifically, in hypopharyngeal carcinoma tissues and adjacent normal tissues, the correlation coefficients of tsRNAs before and after enzymatic treatment were 0.33 and 0.22, respectively, suggesting that tsRNAs carry more RNA modifications, resulting in lower detection efficiency without enzymatic treatment. In contrast, the correlation coefficients of miRNAs before and after enzymatic treatment were 0.63 and 0.58, respectively, indicating that miRNA expression remained relatively stable and was less affected by modifications. This is consistent with the fact that miRNAs typically possess 5′-P and 3′-OH termini and carry fewer modifications than tsRNAs. These results further confirm that PANDORA-seq technology can effectively remove RNA modifications and significantly enhance the detection sensitivity of tsRNAs and rsRNAs, thereby providing a more comprehensive sncRNA tumor expression profile in tumors than traditional RNA-seq.

### 2.2. Dynamic Changes in the Expression and Sequence Characteristics of sncRNA in Hypopharyngeal Carcinoma Tissues and Adjacent Normal Tissues

**Figure 2:**
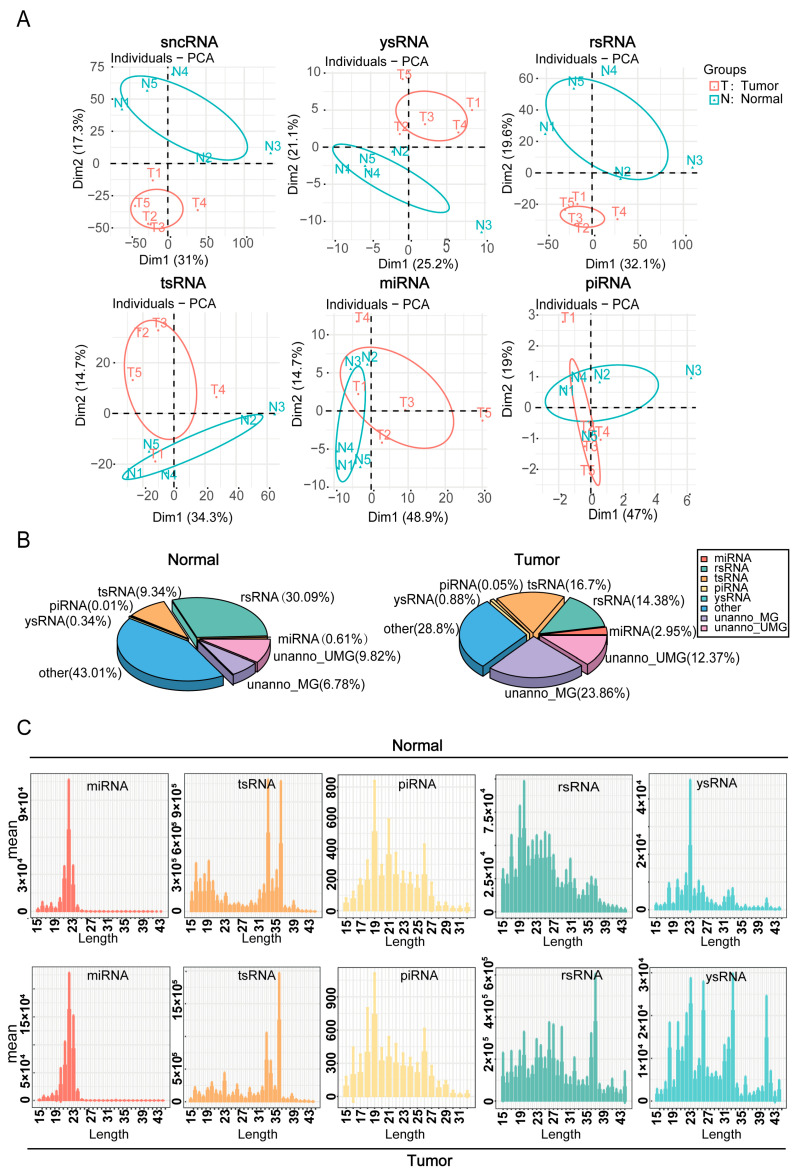
Dynamic changes in sncRNA expression and sequence features. (**A**) Principal component analysis (PCA) based on the read counts of different sncRNA types in the samples. (**B**) Distribution of different sncRNA types in cancerous and adjacent normal tissue samples. (**C**) Length distribution of each sncRNA type in cancerous and adjacent normal tissue samples.

The expression differences and sequence characteristics of sncRNAs in hypopharyngeal cancerous and adjacent normal tissues were evaluated using principal component analysis of sncRNAs and their subclasses (Figure 2A). The results demonstrated significant disparities in the expression patterns of sncRNAs between cancerous and adjacent normal tissues, suggesting that sncRNAs may contribute to the pathogenesis and progression of hypopharyngeal carcinoma. Subsequent analysis of the proportion distribution of different sncRNA types in cancerous and adjacent normal tissues was used to construct the corresponding sncRNA expression profiles (Figure 2B). The data revealed that the expression levels of tsRNA and miRNA were significantly elevated in cancerous tissues, comprising 16.7% and 2.95%, respectively, compared with 9.34% and 0.61% in adjacent normal tissues, respectively. Conversely, rsRNA expression was lower in cancerous tissues (14.38%) than in adjacent normal tissues (30.09%). We also examined the nucleotide length distribution of various sncRNAs (Figure 2C). miRNA peaked at 19–23 nt, and piRNA at 19 nt. In cancerous tissues, tsRNA peaked at 36 nt (vs. 33 nt in adjacent normal tissues). Similarly, rsRNA and ysRNA distributions varied significantly between cancerous and adjacent normal tissues. Collectively, these findings indicate that the expression levels and sequence characteristics of different sncRNA types differed markedly between cancerous and adjacent normal tissues, with miRNA, tsRNA, and rsRNA likely playing key roles in the pathogenesis and progression of hypopharyngeal cancer.

### 2.3. Expression Profiles of rsRNA and tsRNA in Hypopharyngeal Carcinoma Tissues and Adjacent Normal Tissues

**Figure 3:**
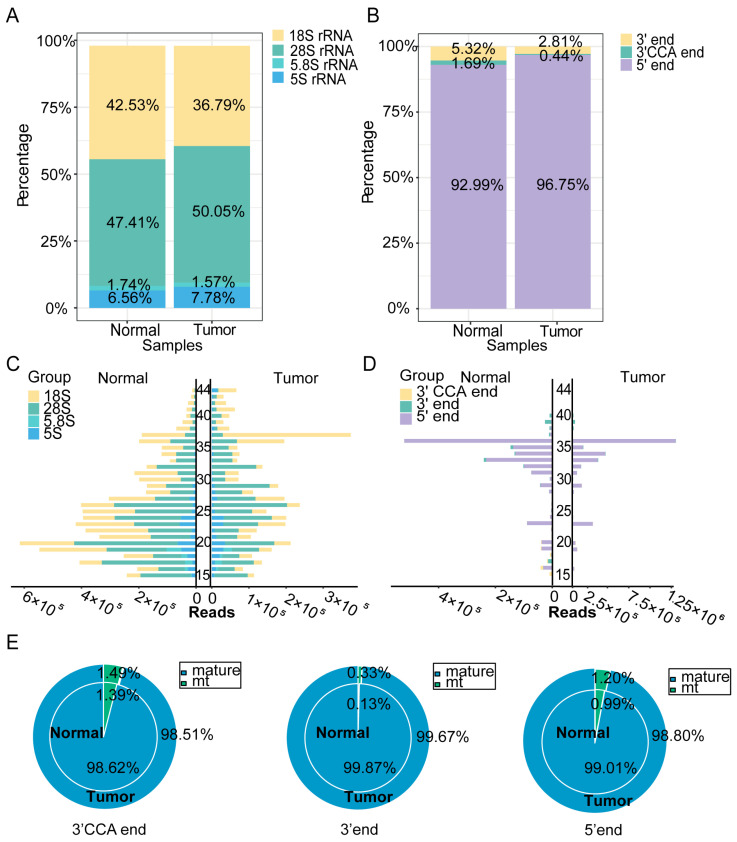
sncRNA profiles of rsRNA and tsRNA in hypopharyngeal carcinoma tissues and adjacent normal tissues. (**A**) Expression profiles of rsRNA derived from different length fragments in cancerous and adjacent normal tissues. (**B**) Expression profiles of various tsRNA types in cancerous and adjacent normal tissues. (**C**) Length distribution of rsRNA subtypes. (**D**) Length distribution of tsRNA subtypes. (**E**) Distribution of different tsRNA types by origin in cancerous and adjacent normal tissues.

The rsRNA and tsRNA expression profiles were further assessed in cancerous and adjacent normal tissues by analyzing their subtypes. Figure 3A shows that the proportion of rsRNA derived from 28S rRNA was higher in cancerous tissues than in adjacent normal tissues, while that from 18S rRNA was lower. Further analysis of the distribution of tsRNA fragments derived from different tRNAs (Figure 3B) revealed that the proportion of 5′-tsRNA was higher in cancerous tissues than in adjacent normal tissues, whereas the proportion of 3′-tsRNA and 3′-CCA tsRNA was relatively lower. These findings suggest that different types of tsRNA may perform distinct biological functions in the development and progression of hypopharyngeal cancer. We also visualized the length distribution of the rsRNA and tsRNA sequences (Figure 3C,D), which showed distinct differences between cancerous and adjacent normal tissues. The analysis of tRNA origins (pre-tRNA, mature tRNA, and mitochondrial tRNA) revealed no significant differences in 3′-CCA tsRNA, 3′-tsRNA, and 5′-tsRNA levels between cancerous and adjacent normal tissues (Figure 3E). Notably, pre-tRNA, due to its relatively low abundance, is not included in Figure 3E. These results collectively indicate that the origins and length distributions of rsRNA and tsRNA differ between cancerous and adjacent normal tissues, suggesting their potential involvement in the regulation of hypopharyngeal cancer development.

### 2.4. Identification of Differentially Expressed sncRNAs

**Figure 4:**
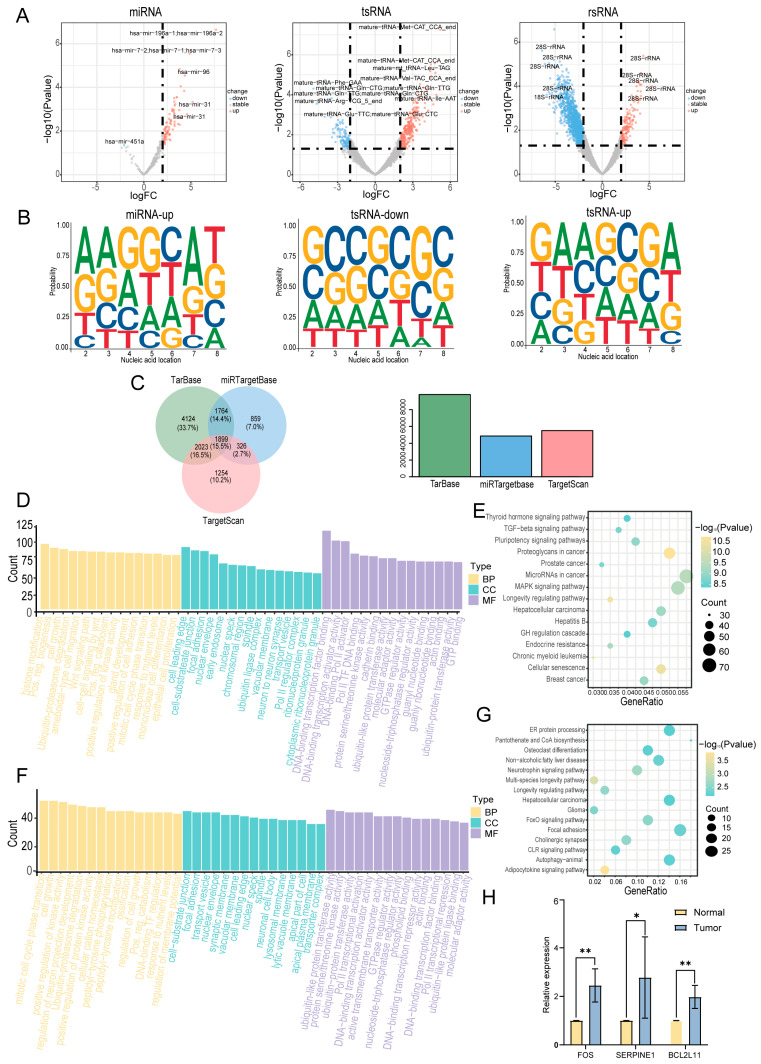
Identification of differentially expressed sncRNAs. (**A**) Volcano plot of sncRNAs differentially expressed between cancerous and adjacent normal tissues. (**B**) Base preference of seed sequences in differentially expressed miRNAs and tsRNAs. (**C**) Venn diagram of predicted target genes of differentially expressed miRNAs in three databases. (**D**) Gene Ontology (GO) enrichment analysis of predicted target genes of miRNAs. Only the top 15 enriched terms by gene count are shown for each category. (**E**) Kyoto Encyclopedia of Genes and Genomes (KEGG) pathway analysis of predicted target genes of miRNAs. Only the top 15 pathways with the most significant differences are shown. (**F**) GO enrichment analysis of predicted target genes of tsRNAs. Only the top 15 enriched terms by gene count are shown for each category. (**G**) KEGG pathway analysis of predicted target genes of tsRNAs. Only the top 15 pathways with the most significant differences are shown. (**H**) Expression of genes related to the MAPK, TGF−β, and FoxO signaling pathways in hypopharyngeal cancer tissues and adjacent normal tissues (* *p* < 0.05; ** *p* < 0.01).

In this study, we employed the R package DESeq2 (version 1.48.1; https://bioconductor.org/packages/release/bioc/html/DESeq2.html, accessed on 20 August 2024) to identify differentially expressed sncRNAs in cancerous tissues compared with adjacent normal tissues. A total of 4798 differentially expressed sncRNAs were identified using the criteria of |log_2_FC| > 1 and *p* < 0.05 (Supplementary Table S2). A volcano plot was constructed to visualize these differentially expressed sncRNAs (Figure 4A). sncRNAs that were significantly upregulated in cancer tissues are highlighted in red, whereas those that were significantly downregulated are highlighted in blue. The top five most significantly upregulated and downregulated sncRNAs were selected for further analysis (Supplementary Table S3). Considering that the functions of miRNAs and tsRNAs are highly dependent on their 5′-seed sequences, we further investigated the nucleotide preferences of these sequences (Figure 4B). The results revealed significant differences in the nucleotide composition of miRNA and tsRNA seed sequences between cancerous and adjacent normal tissues. However, owing to the limited number of miRNAs downregulated in the cancerous tissues, their nucleotide preferences were not included in the analysis. The potential regulatory roles of the differentially expressed miRNAs were assessed by predicting their target genes. A total of 12,249 experimentally validated target genes were identified, of which 1899 were documented in all three databases (TarBase, miRTarBase, and TargetScan) (Figure 4C). Subsequently, we performed Gene Ontology (GO) and Kyoto Encyclopedia of Genes and Genomes (KEGG) functional enrichment analyses. The GO analysis revealed that the target genes of miRNAs were primarily involved in biological processes, including cell development, cell proliferation, and transcriptional regulation (Figure 4D). KEGG analysis revealed that these target genes were primarily enriched in pathways related to cell proliferation and differentiation, including the MAPK and TGF-β signaling pathways (Figure 4E). Similarly, we predicted tsRNA target genes and performed GO and KEGG analyses. The GO analysis revealed that the target genes of tsRNAs were primarily enriched in biological processes, including cell proliferation, cell transport, and protein function (Figure 4F). KEGG analysis revealed that these target genes were primarily enriched in signaling pathways related to apoptosis and cellular metabolism, such as the FoxO signaling pathway (Figure 4G). To further investigate the activation of the key signaling pathways involved in hypopharyngeal carcinoma, we examined the expression levels of genes associated with the MAPK, TGF, and FoxO pathways in hypopharyngeal carcinoma tissues and adjacent non-tumor tissues using qPCR. The results showed that the expression levels of FOS (an MAPK pathway-related gene), SERPINE1 (a TGF-β pathway-related gene), and BCL2L11 (a FoxO pathway-related gene) were significantly higher in hypopharyngeal carcinoma tissues than in adjacent non-tumor tissues (Figure 4H). These findings provide additional evidence supporting the activation of the MAPK, TGF, and FoxO signaling pathways in hypopharyngeal carcinoma. Collectively, these findings suggest that miRNAs and tsRNAs may play crucial roles in the development and progression of hypopharyngeal cancer by regulating key signaling pathways.

### 2.5. Validation of Differentially Expressed sncRNAs

**Figure 5:**
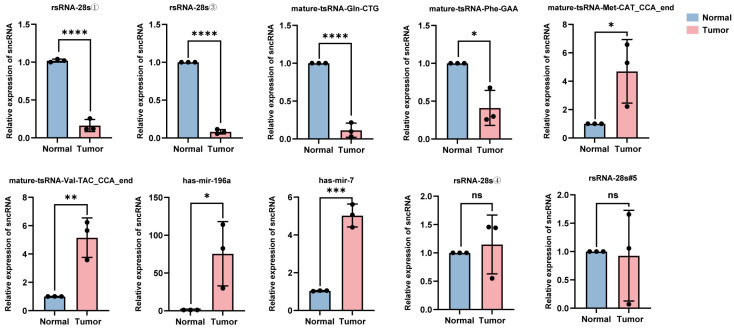
Expression levels of 10 candidate sncRNAs in the cancerous and adjacent normal tissues of stage IV patients. For each sncRNA, three data points are shown, corresponding to three independent replicate experiments (* *p* < 0.05; ** *p* < 0.01; *** *p* < 0.001; **** *p* < 0.0001; ns (*p* > 0.05)).

The total RNA was extracted from the tumor tissues and adjacent normal tissues of five patients with hypopharyngeal carcinoma. Ten sncRNAs that exhibited significant differential expression in tumor tissues and were among the top five candidates identified in the previous screening were randomly selected for validation using RT-qPCR. The relative quantification results showed that compared with adjacent tissues, in the cancer tissues of stage IV patients (i.e., patients 1–3), the expression levels of four sncRNAs (rsRNA-28①, rsRNA-28s③, tsRNA-Gln-CTG, and tsRNA-Phe-GAA) were significantly downregulated, while the expression levels of tsRNA-Met-CAT, tsRNA-Val-TAC, miRNA-196a, and miRNA-7 were significantly upregulated (Figure 5); this was consistent with the trend observed in the sequencing data. However, the broad length range of rsRNA sequences (15–45 nt) and extensive sequence overlap among most rsRNAs limited the precise quantification of target rsRNA expression levels using RT-qPCR. Consequently, no significant upregulation of the rsRNAs was identified in tumor tissues (rsRNA-28s④ and rsRNA-28s#5). Additionally, it is worth noting that the differential expression of sncRNAs between cancerous and adjacent tissues in stage IV patients (i.e., patients 1, 2, and 3) was more pronounced than that in stage II patients (i.e., patients 4 and 5) (Supplementary Figures S1 and S2). This may be because, as the tumor progresses, the differential expression of sncRNAs is not static but rather exhibits a dynamic trend. However, the eight validated sncRNAs with significant differential expression hold potential as biomarkers and therapeutic targets for hypopharyngeal carcinoma.

## 3. Discussion

Hypopharyngeal carcinoma is a highly aggressive malignancy that severely impacts patient survival and quality of life [1]. Despite advances in surgical resection, radiotherapy, and targeted therapies, which have somewhat improved patient outcomes, the overall 5-year survival rate for hypopharyngeal carcinoma has remained at 30–35% over the past decade, with no significant improvement [6,8]. The primary reason for this is the lack of effective early biomarkers, which results in most patients being diagnosed at an advanced stage with high recurrence rates and distant metastasis. In this study, for the first time, we employed PANDORA-seq technology to systematically analyze the dynamic changes in sncRNAs in hypopharyngeal carcinoma tissues and adjacent normal tissues. We identified a series of sncRNAs with significantly differential expression in hypopharyngeal carcinoma and explored their potential roles in the progression of this malignancy. Our results demonstrated that the expression levels of tsRNAs and miRNAs were significantly upregulated, whereas rsRNA expression was downregulated in hypopharyngeal carcinoma tissues, suggesting that these sncRNAs could exert distinct regulatory functions in the development of hypopharyngeal carcinoma. These findings provide new directions to support the investigation of the molecular mechanisms underlying the pathogenesis of hypopharyngeal carcinoma and offer a theoretical basis for the identification of new biomarkers and therapeutic targets.

sncRNAs function as crucial regulatory molecules, playing significant roles in mRNA stability, translation efficiency, and ribosome biogenesis, and are widely implicated in the occurrence and progression of tumors [27,28,29]. The initial investigation into the role of sncRNAs in tumors originated from chronic lymphocytic leukemia (CLL). Calin et al. discovered that miR-15 and miR-16 were located within a 30 kb deletion region in CLL and were significantly downregulated in this disease, thereby first revealing the abnormal expression of sncRNAs in cancer [30]. Subsequently, an increasing number of studies confirmed that sncRNAs are dysregulated in various malignancies, including colorectal, breast, and lung cancers, indicating their diagnostic and prognostic value [15,31,32,33,34]. For example, in non-small cell lung cancer, tRNA-derived small RNA AStDR-007333 promotes tumor progression by activating the HSPB1/MED29 and ELK4/MED29 axes, achieving an AUC of 0.9379 for distinguishing tumor patients from healthy individuals, thereby indicating its substantial potential as a candidate tumor diagnostic biomarker [14]. In pancreatic cancer, tiRNA-Val-CAC-2 is significantly upregulated in the serum of patients with distant metastasis and is negatively correlated with overall survival, suggesting that it has potential as a prognostic biomarker [17]. Notably, in the current study, tiRNA-Val-CAC was also observed to be significantly upregulated in the tissues of hypopharyngeal carcinoma patients (Supplementary Table S2). This finding suggests that it may be involved in the distant metastasis of hypopharyngeal carcinoma, though its underlying mechanisms warrant further investigation.

Currently, research on sncRNAs in hypopharyngeal carcinoma is still in the early stages, with only a few studies reporting on miRNAs [35,36,37,38,39]. For example, Song et al. found that miR-19a promotes the progression of hypopharyngeal squamous cell carcinoma via the SPHK2/PI3K/AKT axis [37]. The expression profiles of sncRNAs in hypopharyngeal carcinoma remain to be systematically elucidated. In this study, we employed PANDORA-seq technology to reveal that miRNAs, tsRNAs, and ysRNAs were significantly upregulated in hypopharyngeal carcinoma tissues compared with adjacent normal tissues, whereas rsRNA expression was markedly reduced. This finding is consistent with that reported by Xia et al. in the serum of patients with acute myeloid leukemia (AML) [40], suggesting that these sncRNAs may perform distinct regulatory roles during carcinogenesis.

Traditional RNA-seq exhibits significant limitations in detecting sncRNAs, particularly tsRNAs and rsRNAs. Specifically, tsRNAs possess unique terminal structures, including 5′-OH, 3′-P, and 2′,3′-cP [23]. These structures interfere with the ligation of 5′ and 3′ adapters during library construction, thereby compromising detection efficiency. Conversely, sncRNAs are frequently modified with various chemical groups (e.g., m^1^A, m^3^C, m^1^G, and m^2^_2_G) that impede the reverse transcription process during library preparation. Consequently, traditional RNA-seq fails to accurately delineate the true expression profiles of sncRNAs [24,25]. In recent years, several research groups have recognized the limitations of traditional RNA-seq and endeavored to optimize the sequencing workflow to obtain more comprehensive sncRNA expression profiles [41,42,43,44,45,46]. Tao et al. [42] employed a kit to remove terminal and internal methylation modifications from sncRNAs and then sequenced tumor and adjacent normal tissues from four colorectal cancer patients. They identified 58 differentially expressed tsRNAs. Subsequently, they validated the upregulation of 5′tiRNA-His-GTG in tumor tissues using RT-qPCR in 25 paired tissue samples and further found that this tsRNA enhanced colorectal cancer cell proliferation and anti-apoptotic capacity by targeting LATS2 and inhibiting the Hippo signaling pathway. However, despite its relative convenience, the kit suffers from poor stability, limited detection accuracy, and a relatively high cost. These factors pose substantial challenges for large-scale studies and clinical applications.

To overcome the limitations of traditional sequencing methods, we developed a novel sncRNA sequencing platform, PANDORA-seq, and established a robust workflow in our preliminary studies [23]. This technology effectively removes key RNA modifications that interfere with adapter ligation and reverse transcription through the combined action of the Alkb and T4PNK enzymes, thereby enabling the comprehensive profiling of sncRNAs with unique modifications. To date, multiple studies have applied PANDORA-seq to precisely profile sncRNA expression [47,48,49]. For example, Gan et al. utilized this technology to reveal the dynamic changes in sncRNA expression in mouse testis tissue under heat stress, and they successfully identified differentially expressed sncRNAs before and after heat stress, providing critical evidence for studying the decline in male reproductive capacity caused by heat stress [49]. However, PANDORA-seq has not yet been used in human oncological studies. Our study demonstrated the application value of PANDORA-seq in oncological research, revealing distinct sncRNA expression patterns between tumors and adjacent normal tissues. This finding not only provides new research directions and approaches for early cancer diagnosis and targeted therapy based on sncRNAs but also lays a solid foundation for the application of PANDORA-seq in tumor research.

An analysis of nucleic acid length distribution revealed that miRNAs peaked at 20–23 nt, whereas tsRNAs, piRNAs, rsRNAs, and ysRNAs exhibited more complex multi-peak distribution patterns. This distribution was consistent with the findings of Gu et al., who reported similar results in peripheral blood mononuclear cells (PBMCs) from lung cancer patients. Gu et al. [19] employed traditional RNA-seq to analyze the expression profiles of sncRNAs in PBMCs from 36 lung cancer patients, 10 pulmonary tuberculosis patients, and 13 healthy individuals. They found that miRNAs in PBMCs were predominantly distributed between 20 and 24 nt, whereas tsRNAs, rsRNAs, and ysRNAs exhibited distinct peaks. This phenomenon further suggests that different types of sncRNAs may have specific biological functions in various cancer types. Notably, the origins of rsRNAs and tsRNAs differed between hypopharyngeal carcinoma tissues and adjacent normal tissues, suggesting their potential involvement in tumor regulation. For instance, the proportion of 5′-tsRNA is higher in tumor tissues than in adjacent normal tissues, and the proportion of rsRNA derived from 28S rRNA is significantly higher in tumor tissues. This finding contrasts with that reported by Xia et al. in the serum of patients with AML, where rsRNAs derived from 18S rRNA were more abundant, and those derived from 28S rRNA were significantly reduced [40]. This discrepancy may be related to the sncRNA expression profiles of different tumor types or the sequencing techniques. Since our study employed PANDORA-seq, which removes RNA modifications and optimizes library construction to provide a more comprehensive sncRNA expression profile than traditional RNA-seq, future research should further elucidate the specific functions of rsRNAs in hypopharyngeal carcinoma and evaluate their potential as biomarkers.

The MAPK signaling pathway plays a crucial regulatory role in cell proliferation and differentiation and is aberrantly activated in various malignancies [50,51,52]. Xu et al. demonstrated that tRF-Val-CAC-016 is downregulated in gastric cancer tissues. This downregulation affects the MAPK signaling pathway by modulating the expression of CACNA1d, thereby inhibiting the proliferation of gastric cancer cells [53]. In this study, we found that sncRNAs differentially expressed in hypopharyngeal carcinoma were also enriched in the MAPK pathway. Moreover, we observed significant upregulation of the MAPK pathway-associated gene FOS in hypopharyngeal cancer tissues. Previous studies have demonstrated that the overexpression of FOS can promote tumor growth and metastasis [54]. In light of these findings, it is plausible to suggest that sncRNAs may contribute to the development and progression of hypopharyngeal carcinoma by modulating the expression of FOS, thereby mediating the MAPK signaling pathway. Additionally, the FoxO protein family plays a key role in cell growth inhibition, metabolic regulation, and apoptosis [55,56], while the TGF-β signaling pathway is crucial for tumor development and metastasis. TGF-β overexpression can induce epithelial–mesenchymal transition, enhancing the migratory and invasive capabilities of tumor cells [57,58]. For example, Ottaviani et al. reported that miR-100 and miR-125b were induced via the TGF-β signaling pathway in pancreatic ductal adenocarcinoma, thereby promoting EMT and tumorigenesis [59]. Our study also revealed that the sncRNAs differentially expressed in hypopharyngeal carcinoma were enriched in the FoxO and TGF-β signaling pathways. Notably, the expression levels of two pathway-associated genes, BCL2L11 and SERPINE1, were significantly upregulated in cancerous tissues compared to adjacent normal tissues. Given the critical roles these genes play in their respective signaling pathways [60,61], our findings suggest a regulatory potential axis in which sncRNAs may modulate the expression of BCL2L11 and SERPINE1, thereby influencing the activity of the FoxO and TGF-β signaling pathways. This regulatory interplay likely has a significant impact on the progression of hypopharyngeal carcinoma, highlighting the potential of sncRNAs as critical modulators in the pathogenesis of this malignancy. However, the specific molecular mechanisms require further experimental validation.

Liquid biopsy technology has enabled non-invasive cancer detection, with sncRNAs emerging as promising biomarkers due to their tissue specificity and stability [13,20,62]. Studies have shown that tRF-Pro-AGG-004 and tRF-Leu-CAG-002 are significantly upregulated in the sera of pancreatic cancer patients. When combined as a 2-tsRNA signature to distinguish pancreatic cancer patients from healthy individuals, the area under the curve reached 0.94, significantly outperforming traditional carbohydrate antigen 19-9 and carcinoembryonic antigen serum biomarkers [12]. In this study, we validated the differential expression of eight sncRNAs in hypopharyngeal carcinoma tissues, supporting their potential as molecular biomarkers.

We employed PANDORA-seq to systematically analyze sncRNA dynamics in hypopharyngeal carcinoma tissues and adjacent normal tissues and identified a series of differentially expressed sncRNAs. We observed the significant upregulation of tsRNAs and miRNAs and the downregulation of rsRNAs in cancerous tissues, suggesting their potential roles in hypopharyngeal cancer development, cell proliferation, and signaling regulation. Functional enrichment analysis revealed that these sncRNAs were primarily associated with the MAPK, FoxO, and TGF-β pathways, with MAPK pathway activation emerging as a potential driver of cancer progression. Our findings indicate the utility of PANDORA-seq in sncRNA tumor profiling and identify significantly differentially expressed sncRNAs as potential biomarkers for early diagnosis and targeted therapy. However, the present study has several limitations, including a small sample size, lack of functional validation, and absence of clinical correlation analysis. Future research should expand the study cohort and integrate in vivo and in vitro experiments to explore how sncRNAs influence the progression of hypopharyngeal carcinoma by modulating the expression of relevant genes in the MAPK, FoxO, and TGF-β signaling pathways. Such investigations will facilitate the clinical translation of sncRNAs in precision diagnosis and personalized treatment. As sncRNA research and liquid biopsy technologies advance, non-invasive sncRNA-based detection may become a valuable strategy for the early diagnosis and personalized treatment of hypopharyngeal cancer. Moreover, although all hypopharyngeal carcinoma patients included in this study were HPV-negative, we did not investigate the correlation between HPV genes and sncRNA expression in hypopharyngeal carcinoma tissues. However, previous studies have demonstrated that HPV positivity is closely associated with specific gene expression patterns and prognostic features in hypopharyngeal carcinoma [63]. In future research, we plan to expand our sample size to include both HPV-negative and HPV-positive hypopharyngeal carcinoma patients, comparing their gene expression profiles to elucidate the impact of HPV status on the biological behavior and treatment response of hypopharyngeal squamous cell carcinoma.

## 4. Materials and Methods

### 4.1. Sample Collection and Clinical Cohort

The samples used in this study were obtained from patients with hypopharyngeal squamous cell carcinoma (HSCC) admitted to the Department of Otolaryngology, Head and Neck Surgery at the First Affiliated Hospital of Chongqing Medical University from October 2023 to March 2024. We enrolled five male patients with HSCC (aged 52–79 years) who had not received prior treatment and were scheduled for surgery. Tumor and adjacent normal tissues (≥2.0 cm from the tumor margin) were collected from each patient. Tissue samples were immediately placed in an RNA preservation solution within 15 min of excision and then transferred to an ultra-low temperature freezer set to −80 °C overnight until nucleic acid extraction. This study was approved by the Ethics Committee of the First Affiliated Hospital of Chongqing Medical University. Informed consent was obtained from each patient before participation. The clinical characteristics of the patients are summarized in Table 1.

### 4.2. Total RNA Isolation and Quality Control

Total RNA was extracted from HSCC tissues and adjacent normal tissues using a commercial kit (Tiangen, Beijing, China) following the manufacturer’s instructions. RNA concentrations were measured using a Nanodrop spectrophotometer (NanoDrop 2000, Thermo Fisher Scientific, Waltham, MA, USA). RNA quality was assessed using agarose gel electrophoresis (Supplementary Figure S3).

### 4.3. Treatment with Alkb and T4PNK

**Figure 6:**
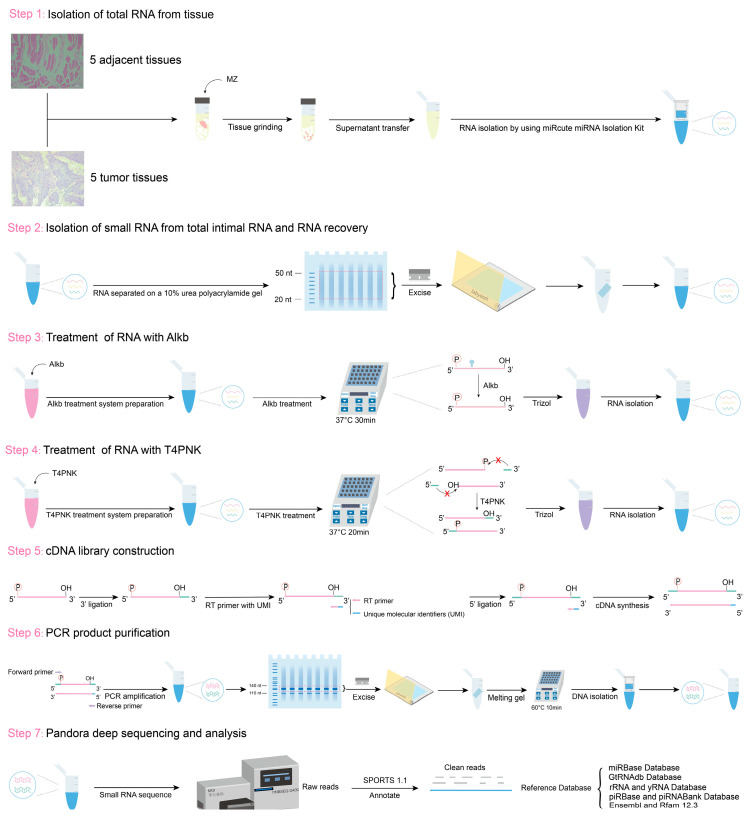
Schematic of RNA sample extraction and PANDORA-seq workflow.

Before Alkb and T4PNK treatment, RNA fragments were separated on a 10% urea polyacrylamide gel, and fragments of 20–50 nucleotides (nt) were recovered. The recovered RNA was treated with Alkb in a 50 μL reaction mixture containing 50 mM HEPES-KOH (pH 8) (Solarbio, Beijing, China), 70 µM ferrous ammonium sulfate (pH 5) (Sigma-Aldrich, St. Louis, MO, USA), 1 mM α-ketoglutarate (Sigma-Aldrich, St. Louis, MO, USA), 2 mM sodium ascorbate (Sigma-Aldrich, St. Louis, MO, USA), 50 mg/L bovine serum albumin (Sigma-Aldrich, St. Louis, MO, USA), 2 µg/µL Alkb, and 1000 U RNase inhibitor (TAKARA, Shigaken, Japan) at 37 °C for 30 min. Subsequently, RNA was extracted using 500 μL of TRIzol reagent (Invitrogen, Carlsbad, CA, USA). The RNA was then treated with T4PNK in a 50 μL reaction mixture containing 10× PNK buffer (NEB, Beverly, MA, USA), 10 mM ATP (NEB, Beverly, MA, USA), and 10 U T4PNK (NEB, Beverly, MA, USA) at 37 °C for 20 min, followed by RNA isolation using 500 μL of TRIzol reagent (Figure 6).

### 4.4. Small RNA Library Construction and Deep Sequencing

Small RNA libraries were constructed using a Small RNA Library Construction Kit (MGI, Shenzhen, China). Initially, a 3′ adapter was ligated to the RNA samples. Specifically, a 5′-adenylated and 3′-blocked single-stranded DNA adapter was ligated to RNA samples. Subsequently, reverse transcription primers containing unique molecular identifiers (UMIs) were introduced to hybridize with the 3′ adapter-ligated RNA and any excess free 3′ adapters. Next, a 5′ adapter was ligated to the 5′ end of the RNA. Following adapter ligation, cDNA synthesis was performed using the UMI-containing reverse transcription primers to extend the RNA templates. The cDNA products were then amplified via PCR using a high-fidelity polymerase targeting the sequences ligated by both the 3′ and 5′ adapters. The amplified library fragments were subsequently size-selected using polyacrylamide gel electrophoresis to isolate PCR products ranging from 110 to 140 bp. Finally, the purified PCR products were denatured and circularized using a circularization primer to form single-stranded circular DNA. Raw sequencing data were generated using the SE 50 mode of the DNBSEQ G400 sequencing platform. The quality of the sequencing data was assessed using FastQC software (version 0.11.9; https://www.bioinformatics.babraham.ac.uk/projects/fastqc/, accessed on 29 October 2024) (Supplementary Figure S4).

### 4.5. Annotation and Analysis of Small RNAs

The small RNA sequences were annotated using SPORTS1.1, a tool specifically designed for annotating small RNAs derived from rRNA and tRNA. Reads were sequentially mapped to the following ncRNA databases: (1) miRbase, (2) GtRNAdb, (3) mitotRNAdb, (4) rRNA and YRNA databases from the National Center for Biotechnology Information, (5) piRBase, and (6) the ncRNAs defined by Ensembl and Rfam 12.3. For tsRNA annotation, tsRNAs were annotated based on pre-tRNA and mature tRNA sequences. Small RNA reads were normalized to reads per million (RPM), and differentially expressed sncRNAs were identified using DESeq2. sncRNAs with log_2_|FC| > 1 and *p* < 0.05 were considered to be significantly differentially expressed.

### 4.6. RT-qPCR

cDNA was synthesized using the HiScript III 1st Strand cDNA Synthesis Kit (Vazyme, Nanjing, China) following the manufacturer’s instructions. The reaction mixture was incubated at 37 °C for 15 min, followed by 5 s of inactivation at 85 °C. The synthesized cDNA was then used to prepare the qPCR mixture and subjected to amplification. The qPCR reaction was conducted using an RT-qPCR machine (Bioer, Hangzhou, China) with the following cycling conditions: initial denaturation at 95 °C for 1 min, 40 cycles of denaturation at 95 °C for 10 s, annealing at 60 °C for 30 s, and extension at 72 °C for 15 s. A melting curve analysis was performed from 72 °C to 95 °C for 5 s. All cDNA samples were normalized using the U6 gene as an internal reference to ensure the accuracy of the experimental results. Each experiment was independently repeated three times to ensure data reliability. The sequences of the primers used are detailed in Supplementary Table S1. Relative expression levels were analyzed using the 2^−ΔΔCt^ method. Statistical analysis was performed using Student’s *t*-test, with *p* < 0.05 considered statistically significant (GraphPad software, version 10.0.2; https://www.graphpad.com/quickcalcs/ttest1/, accessed on 12 March 2025).

## 5. Conclusions

Using PANDORA-seq, we systematically analyzed the sncRNA expression profile of hypopharyngeal carcinoma and identified a series of differentially expressed sncRNAs, including both miRNAs and tsRNAs. These sncRNAs are implicated in key signaling pathways such as the MAPK, FoxO, and TGF-β pathways. The significantly differentially expressed sncRNAs were validated by RT-qPCR and may serve as potential biomarkers and therapeutic targets for hypopharyngeal carcinoma. Our findings provide new directions for research into the molecular mechanisms of this aggressive malignancy and highlight promising avenues for its clinical diagnosis and treatment.

## Figures and Tables

**Table 1:** Clinical information for the patients included in this study.

Patients	Gender	Age (Years)	Tumor Size (mm)	TNM	Metastasis	Type	HPV
1	Male	79	46 × 28 × 50	IVA	Yes	HSCC	Negative
2	Male	67	35 × 20 × 31	IVB	No	HSCC	Negative
3	Male	76	31 × 17 × 32	IVA	Yes	HSCC	Negative
4	Male	52	17 × 18 × 15	II	No	HSCC	Negative
5	Male	70	17 × 16 × 11	II	No	HSCC	Negative

## Data Availability

All data generated or analyzed during this study are included in this published article. The data that support the findings of this study are available from the corresponding author upon request.

## Supplementary Materials

The following supporting information can be downloaded at https://www.mdpi.com/article/10.3390/ijms26135972/s1.

## Abbreviations

The following abbreviations are used in this manuscript:

sncRNAs	Small non-coding RNAs
miRNAs	MicroRNAs
tsRNAs	tRNA-derived small RNAs
rsRNAs	rRNA-derived small RNAs
ysRNAs	YRNA-derived small RNAs
RNA-seq	RNA sequencing
AUC	Area under the curve
PANDORA-seq	Panoramic RNA display by overcoming RNA modification aborted sequencing
Alkb	α-ketoglutarate-dependent hydroxylase
T4PNK	T4 polynucleotide kinase
GO	Gene Ontology
KEGG	Kyoto Encyclopedia of Genes and Genomes
CLL	Chronic lymphocytic leukemia
AML	Acute myeloid leukemia
PBMCs	Peripheral blood mononuclear cells
HSCC	Hypopharyngeal squamous cell carcinoma
UMIs	Unique molecular identifiers
RT-qPCR	Reverse transcription quantitative polymerase chain reaction

## References

[1] Garneau J.C., Bakst R.L., Miles B.A. (2018). Hypopharyngeal cancer: A state of the art review. Oral Oncol..

[2] Aupérin A. (2020). Epidemiology of head and neck cancers: An update. Curr. Opin. Oncol..

[3] Habib A. (2018). Management of advanced hypopharyngeal carcinoma: Systematic review of survival following surgical and non-surgical treatments. J. Laryngol. Otol..

[4] Newman J.R., Connolly T.M., Illing E.A., Kilgore M.L., Locher J.L., Carroll W.R. (2015). Survival trends in hypopharyngeal cancer: A population-based review. Laryngoscope.

[5] Chang J.H., Wu C.C., Yuan K.S., Wu A.T.H., Wu S.Y. (2017). Locoregionally recurrent head and neck squamous cell carcinoma: Incidence, survival, prognostic factors, and treatment outcomes. Oncotarget.

[6] Gambardella C., Polistena A., Sanguinetti A., Patrone R., Napolitano S., Esposito D., Testa D., Marotta V., Faggiano A., Calò P.G. (2017). Unintentional recurrent laryngeal nerve injuries following thyroidectomy: Is it the surgeon who pays the bill?. Int. J. Surg..

[7] Chow L.Q.M., Haddad R., Gupta S., Mahipal A., Mehra R., Tahara M., Berger R., Eder J.P., Burtness B., Lee S.H. (2016). Antitumor Activity of Pembrolizumab in Biomarker-Unselected Patients with Recurrent and/or Metastatic Head and Neck Squamous Cell Carcinoma: Results from the Phase Ib KEYNOTE-012 Expansion Cohort. J. Clin. Oncol. Off. J. Am. Soc. Clin. Oncol..

[8] Fitzmaurice C., Abate D., Abbasi N., Abbastabar H., Abd-Allah F., Abdel-Rahman O., Abdelalim A., Abdoli A., Abdollahpour I., Abdulle A.S.M. (2019). Global, Regional, and National Cancer Incidence, Mortality, Years of Life Lost, Years Lived with Disability, and Disability-Adjusted Life-Years for 29 Cancer Groups, 1990 to 2017: A Systematic Analysis for the Global Burden of Disease Study. JAMA Oncol..

[9] Zhou M., He X., Zhang J., Mei C., Zhong B., Ou C. (2024). tRNA-derived small RNAs in human cancers: Roles, mechanisms, and clinical application. Mol. Cancer.

[10] Di Fazio A., Schlackow M., Pong S.K., Alagia A., Gullerova M. (2022). Dicer dependent tRNA derived small RNAs promote nascent RNA silencing. Nucleic Acids Res..

[11] Zhang B., Chen Z., Tao B., Yi C., Lin Z., Li Y., Shao W., Lin J., Chen J. (2021). m(6)A target microRNAs in serum for cancer detection. Mol. Cancer.

[12] Jin F., Yang L., Wang W., Yuan N., Zhan S., Yang P., Chen X., Ma T., Wang Y. (2021). A novel class of tsRNA signatures as biomarkers for diagnosis and prognosis of pancreatic cancer. Mol. Cancer.

[13] Li K., Lin Y., Luo Y., Xiong X., Wang L., Durante K., Li J., Zhou F., Guo Y., Chen S. (2022). A signature of saliva-derived exosomal small RNAs as predicting biomarker for esophageal carcinoma: A multicenter prospective study. Mol. Cancer.

[14] Yang W., Gao K., Qian Y., Huang Y., Xiang Q., Chen C., Chen Q., Wang Y., Fang F., He Q. (2022). A novel tRNA-derived fragment AS-tDR-007333 promotes the malignancy of NSCLC via the HSPB1/MED29 and ELK4/MED29 axes. J. Hematol. Oncol..

[15] Lu S., Wei X., Tao L., Dong D., Hu W., Zhang Q., Tao Y., Yu C., Sun D., Cheng H. (2022). A novel tRNA-derived fragment tRF-3022b modulates cell apoptosis and M2 macrophage polarization via binding to cytokines in colorectal cancer. J. Hematol. Oncol..

[16] Wang J., Ma G., Ge H., Han X., Mao X., Wang X., Veeramootoo J.S., Xia T., Liu X., Wang S. (2021). Circulating tRNA-derived small RNAs (tsRNAs) signature for the diagnosis and prognosis of breast cancer. NPJ Breast Cancer.

[17] Xiong Q., Zhang Y., Xu Y., Yang Y., Zhang Z., Zhou Y., Zhang S., Zhou L., Wan X., Yang X. (2024). tiRNA-Val-CAC-2 interacts with FUBP1 to promote pancreatic cancer metastasis by activating c-MYC transcription. Oncogene.

[18] Wang Z., Wang H., Zhou S., Mao J., Zhan Z., Duan S. (2024). miRNA interplay: Mechanisms and therapeutic interventions in cancer. MedComm–Oncology.

[19] Gu W., Shi J., Liu H., Zhang X., Zhou J.J., Li M., Zhou D., Li R., Lv J., Wen G. (2020). Peripheral blood non-canonical small non-coding RNAs as novel biomarkers in lung cancer. Mol. Cancer.

[20] Zhu L., Li J., Gong Y., Wu Q., Tan S., Sun D., Xu X., Zuo Y., Zhao Y., Wei Y.Q. (2019). Exosomal tRNA-derived small RNA as a promising biomarker for cancer diagnosis. Mol. Cancer.

[21] Cui H., Li H., Wu H., Du F., Xie X., Zeng S., Zhang Z., Dong K., Shang L., Jing C. (2022). A novel 3′tRNA-derived fragment tRF-Val promotes proliferation and inhibits apoptosis by targeting EEF1A1 in gastric cancer. Cell Death Dis..

[22] Honda S., Loher P., Shigematsu M., Palazzo J.P., Suzuki R., Imoto I., Rigoutsos I., Kirino Y. (2015). Sex hormone-dependent tRNA halves enhance cell proliferation in breast and prostate cancers. Proc. Natl. Acad. Sci. USA.

[23] Shi J., Zhang Y., Tan D., Zhang X., Yan M., Zhang Y., Franklin R., Shahbazi M., Mackinlay K., Liu S. (2021). PANDORA-seq expands the repertoire of regulatory small RNAs by overcoming RNA modifications. Nat. Cell Biol..

[24] Wang H., Huang R., Li L., Zhu J., Li Z., Peng C., Zhuang X., Lin H., Shi S., Huang P. (2021). CPA-seq reveals small ncRNAs with methylated nucleosides and diverse termini. Cell Discov..

[25] Cozen A.E., Quartley E., Holmes A.D., Hrabeta-Robinson E., Phizicky E.M., Lowe T.M. (2015). ARM-seq: AlkB-facilitated RNA methylation sequencing reveals a complex landscape of modified tRNA fragments. Nat. Methods.

[26] Zheng G., Qin Y., Clark W.C., Dai Q., Yi C., He C., Lambowitz A.M., Pan T. (2015). Efficient and quantitative high-throughput tRNA sequencing. Nat. Methods.

[27] Almstrup K., Lobo J., Mørup N., Belge G., Rajpert-De Meyts E., Looijenga L.H.J., Dieckmann K.P. (2020). Application of miRNAs in the diagnosis and monitoring of testicular germ cell tumours. Nat. Rev. Urol..

[28] Shi J., Zhou T., Chen Q. (2022). Exploring the expanding universe of small RNAs. Nat. Cell Biol..

[29] Xiong Q., Zhang Y. (2023). Small RNA modifications: Regulatory molecules and potential applications. J. Hematol. Oncol..

[30] Calin G.A., Dumitru C.D., Shimizu M., Bichi R., Zupo S., Noch E., Aldler H., Rattan S., Keating M., Rai K. (2002). Frequent deletions and down-regulation of micro- RNA genes miR15 and miR16 at 13q14 in chronic lymphocytic leukemia. Proc. Natl. Acad. Sci. USA.

[31] Dhahbi J.M., Spindler S.R., Atamna H., Boffelli D., Martin D.I. (2014). Deep Sequencing of Serum Small RNAs Identifies Patterns of 5′ tRNA Half and YRNA Fragment Expression Associated with Breast Cancer. Biomark. Cancer.

[32] Chu C., Yu L., Wu B., Ma L., Gou L.T., He M., Guo Y., Li Z.T., Gao W., Shi H. (2017). A sequence of 28S rRNA-derived small RNAs is enriched in mature sperm and various somatic tissues and possibly associates with inflammation. J. Mol. Cell Biol..

[33] Wang Q., Song X., Zhao F., Chen Q., Xia W., Dong G., Xu L., Mao Q., Jiang F. (2023). Noninvasive diagnosis of pulmonary nodules using a circulating tsRNA-based nomogram. Cancer Sci..

[34] Bertoli G., Cava C., Castiglioni I. (2015). MicroRNAs: New Biomarkers for Diagnosis, Prognosis, Therapy Prediction and Therapeutic Tools for Breast Cancer. Theranostics.

[35] Lau H.C., Yuan X., Huang H., Zhang M., Hsueh C.Y., Gong H. (2023). Fusobacterium nucleatum facilitates proliferation and autophagy by activating miR-361-3p/NUDT1 axis through oxidative stress in hypopharyngeal squamous cell carcinoma. BMC Cancer.

[36] Li C., Li W., Cao S., Xu J., Qian Y., Pan X., Lei D., Wei D. (2021). Circ_0058106 promotes proliferation, metastasis and EMT process by regulating Wnt2b/β-catenin/c-Myc pathway through miR-185-3p in hypopharyngeal squamous cell carcinoma. Cell Death Dis..

[37] Song J., Li Y., Lu T., Pan M., Wang Z., Liu C., Liao Y., Hu G. (2023). miR-19a mediates the mechanism by which SPHK2 regulates hypopharyngeal squamous cell carcinoma progression through the PI3K/AKT axis. Am. J. Cancer Res..

[38] Xu X., Lu Z., Gross N., Li G., Zhang F., Lei D., Pan X. (2019). A 3-miRNA signature predicts survival of patients with hypopharyngeal squamous cell carcinoma after post-operative radiotherapy. J. Cell. Mol. Med..

[39] Hu Y., He Z., Han B., Lin Z., Zhou P., Li S., Huang S., Chen X. (2024). miR-107 Targets NSG1 to Regulate Hypopharyngeal Squamous Cell Carcinoma Progression through ERK Pathway. Int. J. Mol. Sci..

[40] Xia L., Guo H., Wu X., Xu Y., Zhao P., Yan B., Zeng Y., He Y., Chen D., Gale R.P. (2023). Human circulating small non-coding RNA signature as a non-invasive biomarker in clinical diagnosis of acute myeloid leukaemia. Theranostics.

[41] Zhao R., Yang Z., Zhao B., Li W., Liu Y., Chen X., Cao J., Zhang J., Guo Y., Xu L. (2023). A novel tyrosine tRNA-derived fragment, tRF(Tyr), induces oncogenesis and lactate accumulation in LSCC by interacting with LDHA. Cell. Mol. Biol. Lett..

[42] Tao E.W., Wang H.L., Cheng W.Y., Liu Q.Q., Chen Y.X., Gao Q.Y. (2021). A specific tRNA half, 5′tiRNA-His-GTG, responds to hypoxia via the HIF1α/ANG axis and promotes colorectal cancer progression by regulating LATS2. J. Exp. Clin. Cancer Res..

[43] Fu B., Lou Y., Lu X., Wu Z., Ni J., Jin C., Wu P., Xu C. (2024). tRF-1:30-Gly-CCC-3 inhibits thyroid cancer via binding to PC and modulating metabolic reprogramming. Life Sci. Alliance.

[44] Giraldez M.D., Spengler R.M., Etheridge A., Goicochea A.J., Tuck M., Choi S.W., Galas D.J., Tewari M. (2019). Phospho-RNA-seq: A modified small RNA-seq method that reveals circulating mRNA and lncRNA fragments as potential biomarkers in human plasma. EMBO J..

[45] Maguire S., Lohman G.J.S., Guan S. (2020). A low-bias and sensitive small RNA library preparation method using randomized splint ligation. Nucleic Acids Res..

[46] Wang Y., Katanski C.D., Watkins C., Pan J.N., Dai Q., Jiang Z., Pan T. (2021). A high-throughput screening method for evolving a demethylase enzyme with improved and new functionalities. Nucleic Acids Res..

[47] Liu J., Shi J., Hernandez R., Li X., Konchadi P., Miyake Y., Chen Q., Zhou T., Zhou C. (2023). Paternal phthalate exposure-elicited offspring metabolic disorders are associated with altered sperm small RNAs in mice. Environ. Int..

[48] Hernandez R., Li X., Shi J., Dave T.R., Zhou T., Chen Q., Zhou C. (2024). Paternal hypercholesterolemia elicits sex-specific exacerbation of atherosclerosis in offspring. JCI Insight.

[49] Gan M., Lei Y., Wang K., Wang Y., Liao T., Ma J., Zhu L., Shen L. (2024). A dataset of hidden small non-coding RNA in the testis of heat-stressed models revealed by Pandora-seq. Sci. Data.

[50] Samatar A.A., Poulikakos P.I. (2014). Targeting RAS-ERK signalling in cancer: Promises and challenges. Nat. Rev. Drug Discov..

[51] Ponsioen B., Post J.B., Buissant des Amorie J.R., Laskaris D., van Ineveld R.L., Kersten S., Bertotti A., Sassi F., Sipieter F., Cappe B. (2021). Quantifying single-cell ERK dynamics in colorectal cancer organoids reveals EGFR as an amplifier of oncogenic MAPK pathway signalling. Nat. Cell Biol..

[52] Ravichandran M., Hu J., Cai C., Ward N.P., Venida A., Foakes C., Kuljanin M., Yang A., Hennessey C.J., Yang Y. (2022). Coordinated Transcriptional and Catabolic Programs Support Iron-Dependent Adaptation to RAS-MAPK Pathway Inhibition in Pancreatic Cancer. Cancer Discov..

[53] Xu W., Zheng J., Wang X., Zhou B., Chen H., Li G., Yan F. (2022). tRF-Val-CAC-016 modulates the transduction of CACNA1d-mediated MAPK signaling pathways to suppress the proliferation of gastric carcinoma. Cell Commun. Signal..

[54] Yan S., Zhao W., Du J., Teng L., Yu T., Xu P., Liu J., Yang R., Dong Y., Wang H. (2025). C-FOS promotes the formation of neutrophil extracellular traps and the recruitment of neutrophils in lung metastasis of triple-negative breast cancer. J. Exp. Clin. Cancer Res..

[55] Farhan M., Wang H., Gaur U., Little P.J., Xu J., Zheng W. (2017). FOXO Signaling Pathways as Therapeutic Targets in Cancer. Int. J. Biol. Sci..

[56] Yang X.W., Shen G.Z., Cao L.Q., Jiang X.F., Peng H.P., Shen G., Chen D., Xue P. (2014). MicroRNA-1269 promotes proliferation in human hepatocellular carcinoma via downregulation of FOXO1. BMC Cancer.

[57] Bracken C.P., Goodall G.J., Gregory P.A. (2024). RNA regulatory mechanisms controlling TGF-β signaling and EMT in cancer. Semin. Cancer Biol..

[58] Peng D., Fu M., Wang M., Wei Y., Wei X. (2022). Targeting TGF-β signal transduction for fibrosis and cancer therapy. Mol. Cancer.

[59] Ottaviani S., Stebbing J., Frampton A.E., Zagorac S., Krell J., de Giorgio A., Trabulo S.M., Nguyen V.T.M., Magnani L., Feng H. (2019). Author Correction: TGF-β induces miR-100 and miR-125b but blocks let-7a through LIN28B controlling PDAC progression. Nat. Commun..

[60] Essafi A., Fernández de Mattos S., Hassen Y.A., Soeiro I., Mufti G.J., Thomas N.S., Medema R.H., Lam E.W. (2005). Direct transcriptional regulation of Bim by FoxO3a mediates STI571-induced apoptosis in Bcr-Abl-expressing cells. Oncogene.

[61] Habibzadeh G., Mokhtari K., Heshmati M., Salimy S., Mei Z., Entezari M., Hashemi M., Fu J., Maghsoudloo M. (2025). Identification of lncRNA associated with the SERPINE1 gene in colorectal cancer through TGF-β pathway. Comput. Biol. Med..

[62] Wu Y., Yang X., Jiang G., Zhang H., Ge L., Chen F., Li J., Liu H., Wang H. (2021). 5′-tRF-GlyGCC: A tRNA-derived small RNA as a novel biomarker for colorectal cancer diagnosis. Genome Med..

[63] Pinatti L.M., Walline H.M., Carey T.E. (2018). Human Papillomavirus Genome Integration and Head and Neck Cancer. J. Dent. Res..

