# Supplementary material for: PANDORA-Seq Unveils the Hidden Small Non-Coding RNA Landscape in Hypopharyngeal Carcinoma
Source: Int J Mol Sci. 2025 Jun 21;26(13):5972. doi: 10.3390/ijms26135972 (PMC12250498; doi:10.3390/ijms26135972)
Supplement: Supplementary file 1 [file ijms-26-05972-s001.zip › titles of supplementary files.pdf]

Figure S1: Expression levels of 10 candidate sncRNAs in cancerous and adjacent normal tissues of three stage IV patients. Each panel (A, B, and C) represents the expression profiles of the 10 sncRNAs in one patient. For each sncRNA, three data points are shown, corresponding to three independent replicate experiments. For each sncRNA, three data points are shown, corresponding to three independent replicate experiments (*p<0.05; **p<0.01; ***p<0.001; ****p<0.0001; ns (p > 0.05));

Figure S2: Expression levels of 10 candidate sncRNAs in cancerous and adjacent normal tissues of two stage Ⅱ patients. Each panel (A and B) represents the expression profiles of the 10 sncRNAs in one patient. For each sncRNA, three data points are shown, corresponding to three independent replicate experiments. For each sncRNA, three data points are shown, corresponding to three independent replicate experiments (*p<0.05; **p<0.01; ***p<0.001; ns (p > 0.05));

Figure S3: Agarose gel electrophoresis; Figure S4: Base sequence quality distribution plots.

Table S1: Primer sequences used for reverse transcription (RT) and PCR;

Table S2: Differentially expressed sncRNAs between hypopharyngeal carcinoma tissues and adjacent normal tissues detected by PANDORA-seq;

Table S3: Top five most significantly upregulated and downregulated sncRNAs between hypopharyngeal carcinoma tissues and adjacent normal tissues detected by PANDORA-seq.
