# Supplementary figures and images for: PANDORA-Seq Unveils the Hidden Small Non-Coding RNA Landscape in Hypopharyngeal Carcinoma
Source: Int J Mol Sci. 2025 Jun 21;26(13):5972. doi: 10.3390/ijms26135972 (PMC12250498; doi:10.3390/ijms26135972)

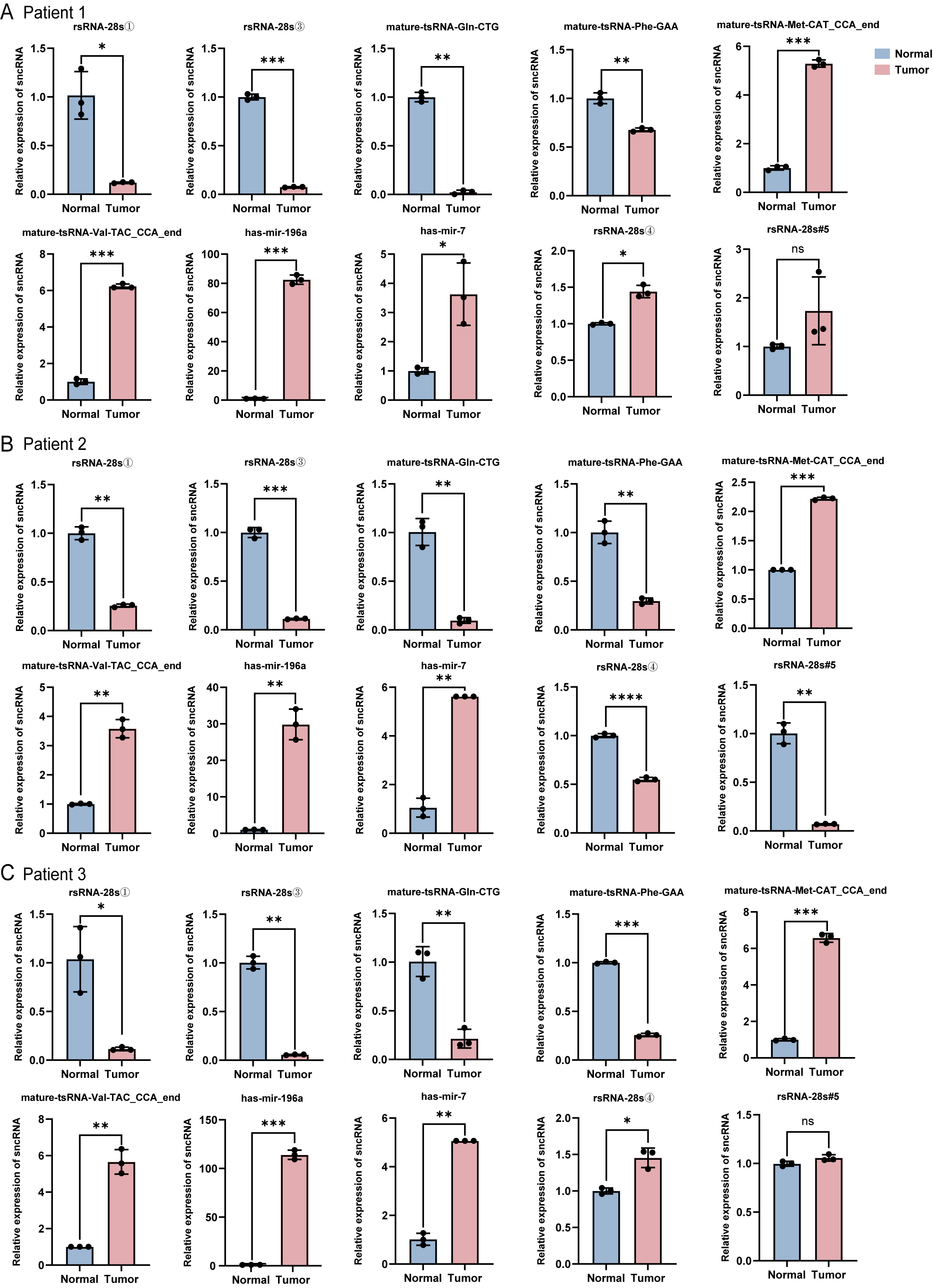

Supplement: Supplementary file 1 [file ijms-26-05972-s001.zip › Figure S1.Expression levels of 10 candidate sncRNAs in cancerous and adjacent normal tissues of three stage IV patients..tif]

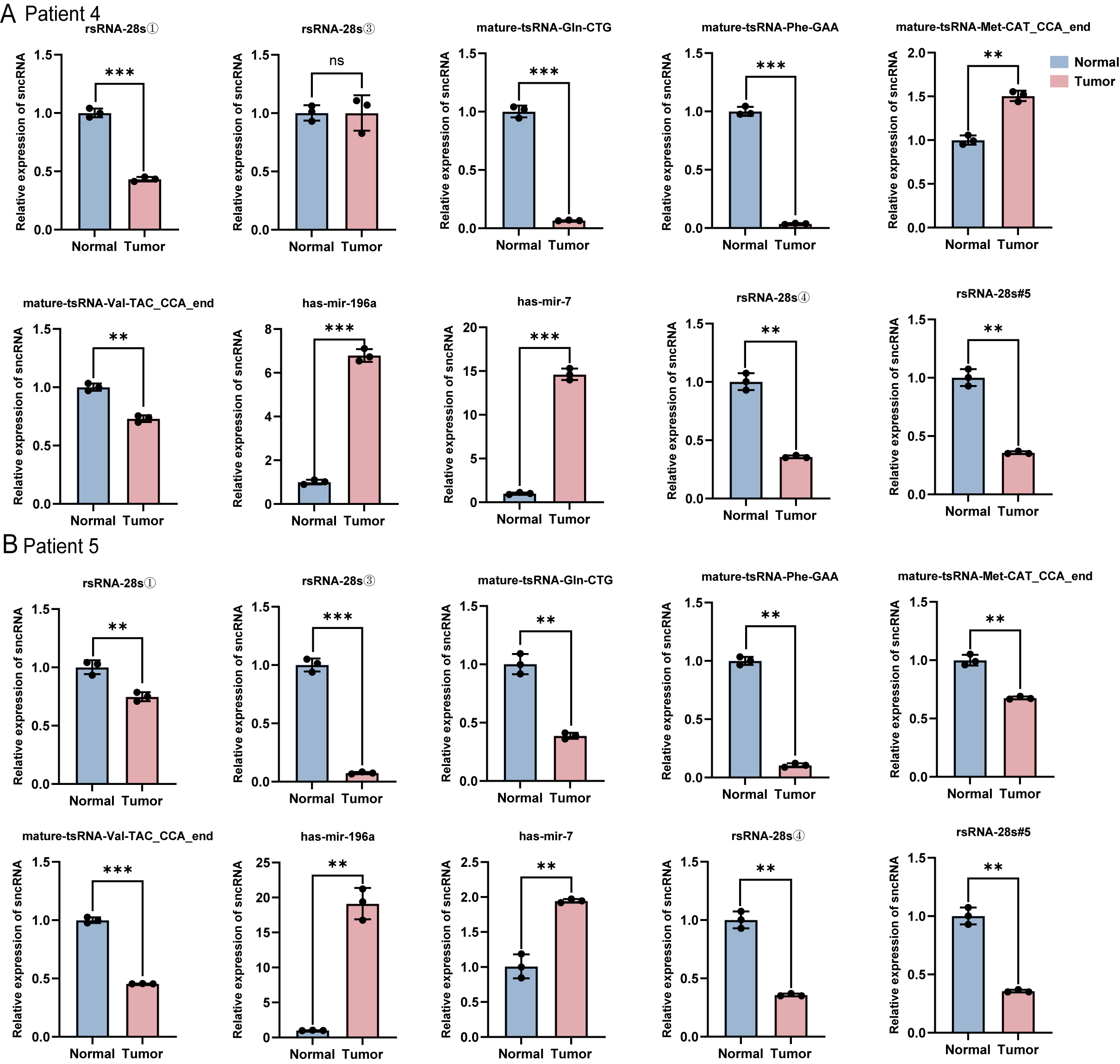

Supplement: Supplementary file 1 [file ijms-26-05972-s001.zip › Figure S2.Expression levels of 10 candidate sncRNAs in cancerous and adjacent normal tissues of two stage II patients..tif]
